# Gender Difference in Associations Between Telomere Length and Risk Factors in Patients With Stroke

**DOI:** 10.3389/fnagi.2021.719538

**Published:** 2021-11-04

**Authors:** Yuqing Wang, Fengjuan Jiao, Huancheng Zheng, Qingsheng Kong, Ran Li, Xiaojie Zhang, Li Yan, Yanlei Hao, Yili Wu

**Affiliations:** ^1^Cheeloo College of Medicine, Shandong University, Jinan, China; ^2^Shandong Collaborative Innovation Center for Diagnosis, Treatment and Behavioral Interventions of Mental Disorders, Institute of Mental Health, Jining Medical University, Jining, China; ^3^Collaborative Innovation Center for Birth Defect Research and Transformation of Shandong Province, Jining Medical University, Jining, China; ^4^Department of Psychiatry, The Second Xiangya Hospital, Central South University, Changsha, China; ^5^National Clinical Research Center for Mental Disorders, Changsha, China; ^6^Department of Neurology, China-Japan Friendship Hospital, Beijing, China; ^7^Department of Neurology, Affiliated Hospital of Jining Medical University, Jining, China; ^8^Key Laboratory of Alzheimer’s Disease of Zhejiang Province, Institute of Aging, School of Mental Health, Wenzhou Medical University, Wenzhou, China; ^9^The Affiliated Kangning Hospital, Wenzhou Medical University, Wenzhou, China; ^10^Oujiang Laboratory, Wenzhou, China; ^11^Shandong Collaborative Innovation Center for Diagnosis, Treatment and Behavioral Interventions of Mental Disorders, Institute of Mental Health, Jining Medical University, Jining, China

**Keywords:** gender, risk factors, stroke, telomere length, gender difference

## Abstract

Multiple risk factors of stroke are associated with telomere length shortening. Although leukocyte telomere length (LTL) is shorter in patients with stroke, the heterogeneity is high. Risk factors may be differentially associated with LTL in male and female patients contributing to the heterogeneity. However, the gender difference in associations between LTL and risk factors in stroke patients has not been investigated. In this study, we investigated the gender difference in associations between LTL and risk factors in 312 stroke patients. Real-time quantitative PCR was used to determine relative LTL, and multiple linear regression analysis was applied for association analyses. We found that LTL was negatively associated with triglyceride (TG) in all patients [β(95% CI) = −0.69 (−1.26, −0.11), *P* < 0.05] after adjusting confounders. Importantly, LTL was negatively associated with lack of exercise [β(95% CI) = −1.80 (−3.12, −0.49), *P* < 0.05] and LDL levels [β(95% CI) = −3.22 (−6.05, −0.390), *P* < 0.05] in male patients, while LTL was negatively associated with dyssomnia [β(95%CI) = −2.00 (−3.96, −0.07), *P* < 0.05] and diabetes [β(95%CI) = −2.13 (−4.10, −0.27), *P* < 0.01] in female patients. Our study showed that LTL is differently associated with risk factors in male and female patients with stroke, indicating that gender difference should be considered when LTL is potentially applied as an index of risk and prognosis for stroke. Our study also provides an insight into that gender differences should be considered when developing intervention strategies for stroke prevention and treatment.

## Introduction

Stroke, an age-related disease, is one of the leading causes of death and long-term disability worldwide, bringing a heavy burden to both family and society (Donnan et al., 2008). As an age-related disease, the risk of stroke is increased with age (Calado and Young, 2009). In addition, risk factors including current smoking, alcohol intake, unhealthy diet, abdominal obesity, less physical activity, hypertension, and diabetes mellitus, etc., are associated with a 90% risk of stroke and prognosis of stroke (O’Donnell et al., 2010). Telomere length is declined with age in adults less than 75 years (Lapham et al., 2015). Overall telomere attrition predicts mortality and risk of aging-related diseases in general human cohorts (Blackburn et al., 2015). Consistently, leukocyte telomere length (LTL) shortening was associated with the risk of stroke (Kappei and Londoño-Vallejo, 2008; Luo et al., 2017; Gao et al., 2018; Jin et al., 2018; Tian et al., 2019). Moreover, stroke-related risk factors such as obesity, smoking, alcohol intake, psychological stress, and depression are linked to telomere length shortening (Tian et al., 2019). However, telomere length shortening does not have a causal effect on stroke based on Mendelian randomization studies (Cao et al., 2019, 2020). It suggests that LTL shortening might be the consequence of the pooled effect of risk factors. Thus, LTL shortening may serve as an index of stroke risk and potentially serve as an index of prognosis for stroke.

The heterogeneity of LTL is high in various studies, although LTL is short in patients with stroke in a meta-analysis (Jin et al., 2018; Tian et al., 2019). Gender difference may be a key factor for the heterogeneity as a gender-based difference of LTL is observed in the general population (Gardner et al., 2014; Needham et al., 2014; Lapham et al., 2015; Choi et al., 2018; Ghimire et al., 2019). Moreover, gender-specific associations between LTL and multiple factors including risk factors have been observed in the general population or in patients with coronary artery disease, an age-related disease (Cheng et al., 2017; Shadyab et al., 2017a, b; Ghimire et al., 2019; Sullivan et al., 2019). However, none of the aforementioned studies examined associations between LTL and risk factors in male and female stroke patients, respectively. In this study, we aimed to explore differential associations between risk factors and LTL in male and female stroke patients, respectively. It may provide an insight into that gender difference should be considered when LTL is potentially applied as an index of risk and prognosis for stroke and that gender difference should be considered when developing intervention strategies for stroke prevention and treatment.

## Subjects and Methods

### Participants

Three hundred fifty-one patients were enrolled from the Affiliated Hospital of Jining Medical University between May 2016 and February 2017; 39 patients were excluded because of missing information or not meeting the inclusion criteria. Thus, 312 stroke patients were included in the final data analysis. Inclusion criteria: patients diagnosed with ischemic stroke (ICD-10: 163.9) or diagnosed with temporary ischemic attacks (TIAs, ICD-10: G45.9) based on “Chinese guidelines for diagnosis and treatment of acute ischemic stroke (2010)”; the first-onset stroke or TIAs; and patients with consciousness. Exclusion criteria: patients with unconscious disorders, language disorders, dementia, neuropsychiatric, or severe complications. This is a cross-sectional study. Experiments were conducted according to the principles expressed in the Declaration of Helsinki. This study was approved by the Research Ethics Committee of Jining Medical University. The written informed consents were obtained from patients or their guardians.

### Data Collection

On the day of admission, the clinical data were collected by neurologists. The information of demographic characteristics, lifestyles, and disease history was collected and shown in Table 1. Subjects who smoked more than one cigarette/day for 1 year or above were defined as current smokers. Smokers who had quit smoking for more than half a year were defined as former smokers. Subjects who never smoke were defined as non-smokers. Subjects who drank more than one time/week for at least half a year were defined as current alcohol drinkers. Otherwise, subjects were defined as non-alcohol drinkers or occasional alcohol drinkers. Subjects who did physical exercise > 30 min/day were defined as exercise well. The quality of sleep was assessed according to “Guidelines for the diagnosis and treatment of adult insomnia in China (2017).” The patients with the following symptoms were diagnosed with dyssomnia: sleep latency of more than 30 min, ≥ 2 awakenings throughout the night, early-morning awakening, reduced sleep quantity, and/or less than 6.5 h sleep per night, as well as accompanied by excessive sleepiness during the daytime. The medication was collected and presented in Supplementary Table 1. Body mass index (BMI) was calculated as weight divided by squared height. The blood samples were collected within 72 h since the onset of stroke. Specimens were tested on a Roche cobas 8000 system (c701 and c702 modules). The following biochemical indexes, known as risk factors of stroke, were included for data analyses: fasting blood glucose (FBG; normal range value: 3.89 ∼ 6.1 mmol/L); triglyceride (TG; normal range value: 0.45 ∼ 1.69 mmol/L); total cholesterol (TC; normal range value: 3.00 ∼ 5.69 mmol/L); high-density lipoprotein: (HDL; normal range value: 0.70 ∼ 2.00 mmol/L); low-density lipoprotein (LDL; normal range value:<3.12 mmol/L); and very low-density lipoprotein (VLDL; normal range value: 0.21 ∼ 0.78 mmol/L). The data of biochemical indexes were presented in Table 1.

**TABLE 1:** Characteristics of 312 stroke patients.

Variables	Total (*n* = 312) number (%) or mean (SD)	Male (*n* = 208) number (%) or mean (SD)	Female (*n* = 104) number (%) or mean (SD)	*P* (male vs. female)
**Age (years)**				
Total	62.02 (10.70)	60.27 (10.33)	65.52 (10.61)	<0.001**
≤40	1 (0.32)	1 (0.48)	0	
41–50	50 (16.03)	41 (19.71)	9 (8.65)	
51–60	91 (29.17)	68 (32.69)	23 (22.12)	
61–70	99 (31.73)	61 (29.33)	38 (36.54)	
71–80	58 (18.59)	34 (16.35)	24 (23.08)	
81–90	13 (4.17)	3 (1.44)	10 (9.62)	
**Education**				
Never	78 (25.00)	23 (11.06)	55 (52.88)	<0.001**
Secondary education	168 (53.85)	125 (60.10)	43 (41.35)	
High education	66 (21.15)	60 (28.85)	6 (5.77)	
**Smoking status**				
Never smoker	151 (48.40)	53 (25.48)	98 (94.23)	<0.001**
Former smoker	34 (10.90)	34 (16.35)	0	
Current smoker	127 (40.71)	121 (58.17)	6 (5.77)	
**Alcohol drinking status**				
Never/occasionally alcohol drinkers	223 (71.47)	120 (57.69)	99 (95.19)	<0.001**
Alcohol drinker	89 (28.53)	84 (40.38)	5 (4.81)	
**Sleeping status**				
Well	253 (81.09)	179 (86.06)	74 (71.15)	0.002*
Insomnia	59 (18.91)	29 (13.94)	30 (28.85)	
**Exercise habit**				
Well	259 (83.01)	171 (82.21)	88 (84.62)	0.594
Poor	53(16.99)	37 (17.79)	16 (15.38)	
**BMI (kg/m^2^)**				
<24	120 (38.46)	74 (35.58)	46 (44.23)	0.217
24–28	119 (38.14)	86 (41.35)	33 (31.73)	
>28	73 (23.40)	48 (23.08)	25 (24.04)	
FBG (mmol/L)	6.19 (2.50)	6.01 (2.44)	6.57 (2.59)	0.071
TG (mmol/L)	1.59 (1.17)	1.52 (1.24)	1.73 (0.99)	0.144
TC (mmol/L)	4.42 (1.19)	4.34 (1.22)	4.58 (1.12)	0.113
HDL (mmol/L)	1.11 (0.26)	1.09 (0.25)	1.15 (0.27)	0.061
LDL (mmol/L)	2.60 (0.75)	2.59 (0.76)	2.62 (0.75)	0.689
VLDL (mmol/L)	0.71 (0.60)	0.66 (0.64)	0.81 (0.48)	0.038*
**Presence of hypertension**				
No	150 (48.08)	109 (52.40)	41 (39.42)	0.031*
Yes	162 (51.92)	99 (47.60)	63 (60.58)	
**Presence of coronary heart disease**				
No	261 (83.65)	178 (85.58)	83 (79.81)	0.194
Yes	51 (16.35)	30 (14.42)	21 (20.19)	
**Presence of diabetes**				
No	241 (77.24)	168 (80.77)	73 (70.19)	0.036*
Yes	71 (22.76)	40 (19.23)	31 (29.81)	

FBG, fasting blood glucose; TG, triglyceride; TC, total cholesterol; HDL, high-density lipoprotein; LDL, low-density lipoprotein; VLDL, very low-density lipoprotein. *P < 0.05; **P < 0.001.

### The Relative Telomere Length Measurement

Genomic DNA was extracted from whole blood samples using the RelaxGene Blood DNA System (Tiangen Biotech, Co., Ltd., Beijing, China) according to the instructions of the manufacturer and stored in a –80°C freezer (Genetic Resource Center of Jining Medical University). Relative LTL was measured by real-time quantitative PCR (qPCR) (Cawthon, 2002). The gene 36B4 was used as a single copy reference gene to normalize the quantity of input DNA. The cycle threshold (Ct) values were used to calculate the relative telomere length. The equation was *T*/*S* = 2^–(ΔCt)^, where *T* = telomere repeat copy number, *S* = 36B4 copy number, and ΔCt = Ct-telomere—Ct-36B4. All *T*/*S* ratios of telomere DNA were normalized to the *T*/*S* ratio of reference DNA. The primer sequences (5′→3′) were te11b: GGTTTTTGAGGGTGAGGGTGAGGGTGAGGGTGAGGGT, te12b: TCCCGACTATCCCTATCCCTATCCCTATCCCTATCCC TA, 36B4u: CAGCAAGTGGGAAGGTGTAATCC, and 36B4d: CCCATTCTATCATCAACGGGTACAA, respectively. The reaction system consisted of 10 μl of MasterMix, 0.4 μl of each primer, 2 μl of template DNA, and 7.2 μl of H_2_O. Real-time PCR (RT-PCR) was performed using a QuantStudio five Real-Time PCR System with the program of 95°C for 5 min, and 40 cycles of 95°C for 15 s, and 60°C for 1 min. A standard curve was derived from serially diluted reference DNA with good linearity (*R*^2^ > 0.98). Triplicates were applied for each sample, and three or more repetitions were performed for each sample. The assay was repeated when the SD was greater than 0.5 among triplicates.

### Statistical Analysis

Gender-based subgroup analysis was performed. The Kolmogorov–Smirnov goodness-of-fit test was performed to test the normality of continuous data. Continuous data were presented as mean and SD when data were distributed normally, while median and interquartile range (IQR) were presented when data were not distributed normally. Categorical data were presented as frequency. The student’s *t*-test was used for the comparison of normally distributed continuous variables. Mann–Whitney *U* test or Kruskal–Wallis test was used to compare not normally distributed continuous variables. The chi-square test was used for the comparison of frequencies. Fisher’s exact test was used when more than 20% of the cells in any cross-tabulation had an expected count less than or equal to 5. Pearson product-moment correlation analysis was conducted to determine the correlations between LTL and FBG, TG, TC, HDL, or VLDL. Simple linear regression was conducted to determine the single explanatory variable. Multiple linear regression was performed to determine the independent variable. The selection of covariates for male and female patients was based on the following criteria: LTL-related variables reported in previous studies and collected in this study were selected by gender; the frequency of exposure was more than 10% after correlation analysis and univariate analysis by gender. The frequencies of smoking and alcohol consumption were less than 10% in female patients. Thus, these two covariates were not included in female patients. Predictors for all stroke patients (Model 1) were adjusted for BMI, smoking status (current smoker or former smoker/non-smoker), alcohol drinking status (current alcohol drinker/non-alcohol drinkers or occasionally alcohol drinker), exercise status (well/poor), dyssomnia (yes/no), HDL, LDL, VLDL, diabetes (yes/no), and hypertension (yes/no). Predictors for male stroke patients (Model 2) were adjusted for BMI, smoking status (current smoker or former smoker/non-smoker), alcohol drinking status (current alcohol drinker/non-alcohol drinkers or occasionally alcohol drinker), dyssomnia (yes/no), TG, HDL, VLDL, diabetes (yes/no), and hypertension (yes/no). Predictors for female stroke patients (Model 3) were adjusted for BMI, exercise status (well/poor), TG, HDL, LDL, and hypertension (yes/no). *P* < 0.05 was considered statistically significant. Bonferroni correction was applied for correlation analysis and simple linear regression analysis and corrected *P*-values were presented. Cohen’s effect size *f*^2^ was calculated for the statistical power of multiple linear regression. IBM SPSS Statistics was used for data analysis.

## Results

### Characteristics of Participants

A total of 312 stroke patients were included in the study. Multiple risk factors of stroke were included based on previous studies. The age of patients ranges from 28 to 90 years with an average age of 62.02 ± 10.70 years. The mean age of female patients was higher than that of male patients (*p* < 0.05), 65.52 ± 10.61 years vs. 60.27 ± 10.33 years (Table 1). Patients aged 50 to 60 years old were the major portion in males accounting for 32.69%. However, patients aged 60 to 70 years old were the major portion in females accounting for 36.54%. Significant differences in education levels, smoking history, drinking status, sleep status, exercise status, hypertension, and diabetes were detected in both male and female groups (*p* < 0.05) (Table 1). Compared with female patients, male patients had more unhealthy drinking and smoking habits, but higher education level and sleep quality. The prevalence of hypertension and diabetes was lower in male patients (Table 1). It indicated that gender difference of multiple factors does exist in stroke patients.

### Differences of Leukocyte Telomere Length Based on the Variables

Leukocyte telomere length was significantly shorter in patients lacking exercise and in patients with low HDL (*P* < 0.05), separately (Table 2). No significant difference was detected in other aspects. The gender-based subgroup analysis was further conducted. LTL was significantly shorter in male patients lacking exercise and with low HDL (*P* < 0.05), separately (Table 2), while LTL was significantly shorter in female patients with dyssomnia (*P* < 0.05). It indicated that LTL may differently associate with risk factors in male and female patients.

**TABLE 2:** LTL in stroke patients.

Variables	Total		Male		Female		*P* (male vs. female)
	Median (IQR)	*P*	Median (IQR)	*P*	Median (IQR)	*P*	
	2.85 (1.76, 2.69)		2.84 (1.65, 4.75)		2.84 (1.84, 7.88)		0.279
**Age (years)**							
≤40	6.62	0.757	6.62	0.762	-	0.851	-
41–50	2.81 (1.59, 4.29)		2.80 (1.51, 4.13)		2.83 (1.71, 6.53)		0.673
51–60	3.05 (1.77, 6.42)		3.13 (1.73, 6.39)		2.60 (1.77, 7.57)		0.848
61–70	3.05 (1.86, 6.42)		2.77 (1.68, 5.09)		3.35 (2.11, 7.15)		0.221
71–80	2.73 (1.78, 4.74)		2.83 (1.62, 4.20)		2.71 (1.79, 7.71)		0.705
81–90	7.70 (1.13, 10.76)		4.50		8.65 (1.08, 11.05)		0.811
**Education**							
Never	3.22 (1.65, 8.84)	0.170	2.79 (1.60, 7.32)	0.575	3.38 (1.79, 8.97)	0.274	0.588
Secondary education	3.00 (1.79, 2.16)		3.05 (1.74, 5.66)		2.83 (1.90, 7.57)		0.659
High education	2.53 (1.63, 3.92)		2.55 (1.64, 4.19)		2.40 (1.20, 2.98)		0.330
**Smoking status**							
Never smokers	2.83 (1.79, 6.77)	0.827	4.64 (1.67, 6.22)	0.976	4.89 (1.82, 7.62)	0.343	0.651
Former smokers	3.11 (1.64, 4.53)		3.11 (1.64, 4.53)		-		-
Current smokers	2.85 (1.66, 6.26)		2.81 (1.65, 4.97)		7.55 (2.01, 9.55)		0.180
**Alcohol drinking status**							
Never or occasionally alcohol drinkers	2.85 (1.79, 7.18)	0.189	2.90 (1.72, 6.49)	0.260	2.84 (1.84, 7.80)	0.518	0.754
Alcohol drinkers	2.83 (1.62, 4.67)		2.81 (1.61, 4.62)		6.77 (1.83, 12.68)		0.200
**Sleeping status**							
Well	3.05 (1.77, 6.48)	0.098	2.85 (1.68, 4.72)	0.689	3.48 (2.07, 8.70)	0.019*	0.054
dyssomnia	2.52 (1.59, 4.57)		2.81 (1.58, 5.93)		2.27 (1.59, 3.50)		0.660
**Exercise habit**							
Well	3.08 (1.79, 6.99)	0.016*	3.12 (1.76, 6.30)	0.002*	2.98 (1.80, 7.88)	0.698	0.847
Poor	2.40 (1.47, 3.63)		1.86 (1.31, 3.29)		2.83 (2.01, 10.49)		0.053
**BMI**							
<24	3.05 (1.87, 6.38)	0.469	3.12 (1.91, 5.64)	0.153	2.82 (1.79, 7.15)	0.623	0.658
24–28	2.93 (1.58, 6.42)		4.33 (1.42, 6.33)		3.08 (1.84, 7.78)		0.502
>28	2.55 (1.70, 5.90)		2.48 (1.65, 3.57)		3.05 (1.90, 10.59)		0.085
**FBG**							
Normal	2.85 (2.64, 6.62)	0.961	2.83 (1.64, 4.66)	0.64	2.92 (1.64, 8.97)	0.721	0.458
Abnormal	2.84 (1.86, 4.73)		2.74 (1.70, 5.10)		2.95 (2.33, 4.49)		0.225
**TG**							
Normal	3.04 (1.76, 7.16)	0.233	3.05 (1.72, 5.77)	0.151	2.92 (1.78, 9.21)	0.732	0.372
Abnormal	2.83 (1.73, 4.37)		2.81 (1.49, 4.33)		3.08 (2.10, 4.80)		0.164
**TC**							
Normal	2.85 (1.71, 6.26)	0.301	2.85 (1.64, 4.67)	0.694	2.88 (1.83, 8.00)	0.391	0.423
Abnormal	3.37 (1.78, 6.87)		2.93 (1.76, 5.43)		3.50 (2.08, 8.29)		0.363
**HDL**							
Normal	2.95 (1.78, 6.36)	0.009*	3.00 (1.70, 4.86)	0.025*	2.92 (1.88, 7.99)	0.213	0.294
Abnormal	1.66 (1.20, 2.96)		1.69 (1.20, 2.85)		1.64		-
**LDL**							
Normal	2.89 (1.71, 6.33)	0.918	2.84 (1.64, 4.73)	0.964	3.07 (1.83, 7.93)	0.826	0.321
Abnormal	2.82 (1.79, 4.67)		2.93 (1.69, 4.67)		2.82 (2.05, 9.08)		0.634
**VLDL**							
Normal	2.73 (1.68, 5.77)	0.952	2.62 (1.66, 4.64)	0.756	2.73 (1.77, 7.91)	0.847	0.474
Abnormal	3.05 (1.77, 4.74)		3.09 (1.53, 4.61)		2.92 (1.90, 5.65)		0.290
**Presence of hypertension**							
No	2.84 (1.75, 6.73)	0.669	2.81 (1.68, 6.83)	0.351	2.92 (1.89, 6.80)	0.783	0.795
**Presence of coronary heart disease**							
Yes	2.85 (1.74, 5.49)		2.85 (1.62, 4.54)		2.84 (1.79, 8.01)		0.196
No	2.82 (1.68, 5.54)	0.141	2.81 (1.65, 5.54)	0.862	2.82 (1.79, 5.65)	0.051	0.775
Yes	3.72 (1.96, 8.02)		3.54 (1.80, 4.22)		7.80 (2.07, 11.76)		0.072
**Presence of diabetes**							
No	3.12 (1.72, 7.16)	0.140	3.05 (170, 5.37)	0.152	3.38 (1.77, 9.05)	0.424	0.299
Yes	2.81 (1.77, 3.57)		2.72 (1.43, 3.89)		2.82 (2.12, 3.53)		0.348

FBG, fasting blood glucose (normal range value: 3.89 ∼ 6.1 mmol/L) TG, triglyceride (normal range value: 0.45 ∼ 1.69 mmol/L); TC, total cholesterol (normal range value: 3.00 ∼ 5.69 mmol/L); HDL, high-density lipoprotein (normal range value: 0.70 ∼ 2.00 mmol/L); LDL, low-density lipoprotein (normal range value: <3.12 mmol/L); VLDL, very low-density lipoprotein (normal range value: 0.21 ∼ 0.78 mmol/L). *P < 0.05.

Considering medications may contribute to the difference of LTL, the difference of LTL based on medications was analyzed when a medication is taken by more than 10 patients (Zhang et al., 2020; Huang et al., 2021). The medication taken by the patients was summarized in Supplementary Table 1. No difference of LTL was detected based on four medications: calcium channel blocker, biguanides, aspirin, and clopidogrel (Supplementary Table 2). It indicated that medication may have no effect on LTL.

### Associations Between Leukocyte Telomere Length and Multiple Risk Factors

To examine associations between risk factors and LTL, Pearson product-moment correlation analysis and simple linear regression were conducted. The levels of FBG (*r* = −0.115, *P* < 0.05) and TG (*r* = −0.134, *P* < 0.05) were negatively correlated with LTL in all patients. In male patients, the levels of TG (*r* = −0.151, *P* < 0.05) and LDL (*r* = −0.148, *P* < 0.05) were negatively correlated with LTL, separately (Supplementary Table 3). By simple linear regression analysis, LTL was inversely associated with the levels of TG [β(95%CI) = −0.47 (0.86, −0.08), *P* < 0.05] and diabetes [β(95%CI) = −1.28 (−2.34, −0.23), *P* < 0.05] in stroke patients. Importantly, LTL was inversely associated with lack of exercise [β(95%CI) = −1.96 (−3.29, −0.63), *P* < 0.05] and abnormal TG levels [β(95%CI) = −0.46 (−0.88, −0.04), *P* < 0.05] in male patient, while short LTL was associated with diabetes [β(95%CI) = −2.19 (−4.01, −0.38), *P* < 0.05] in female patients (Table 3). The regression coefficients were not significant when a Bonferroni correction was applied (corrected *P* < 0.004).

**TABLE 3:** Simple linear regression analysis for LTL.

Variable	Simple linear regression analysis					
	All (*n* = 312)		Male (*n* = 208)		Female (*n* = 104)	
	β (95% CI)	*P*	β (95% CI)	*P*	β (95% CI)	*P*
Age	0.02 (−0.02, 0.06)	0.413	−0.01 (−0.05, 0.046)	0.847	0.04 (−0.04, 0.12)	0.304
Education	−0.36 (−0.73, 0.02)	0.065	−0.28 (−0.76, 0.19)	0.242	−0.32 (−1.15, 0.51)	0.447
Smoking status	−0.53 (−1.42, 0.36)	0.241	−0.45 (−1.64, 0.74)	0.457	1.46 (−2.20, 5.11)	0.431
Alcohol drinking	−0.48 (−1.40. 0.45)	0.310	−0.39 (−1.48, 0.69)	0.470	2.32 (−1.34, 5.97)	0.211
Dyssomnia	−0.59 (−1.73, 0.55)	0.310	0.09 (−1.42, 1.59)	0.910	−1.75 (−3.60, 0.11)	0.065
Lack of exercise	−1.02 (−2.21, 1.16)	0.090	−1.96 (−3.29, −0.63)	0.004*	1.16 (−1.20, 3.52)	0.331
BMI	0.01 (−0.11. 0.13)	0.880	−0.09 (−2.25, 0.07)	0.260	0.15 (−0.06, 0.35)	0.155
FBG	−0.16 (−0.34, 0.02)	0.089	−0.13 (−0.35, 0.08)	0.223	0.25 (−0.58, 0.09)	0.147
TG	−0.47 (0.86, −0.08)	0.018	−0.46 (−0.88, −0.04)	0.032*	−0.63 (−1.51, 0.25)	0.159
HDL	1.37 (−0.38, 3.12)	0.126	1.41 (−0.68, 3.49)	0.185	0,93 (−2.34, 4.21)	0.573
LDL	−0.50 (−1.11, 0.10)	0.103	−0.74 (−1.43, 0.05)	0.036	−0.04 (−1.24, 1.16)	0.947
VLDL	−0.09 (−0.81, 0.64)	0.819	−0.24 (−1.03, 0.55)	0.550	0.17 (−1.60, 1.94)	0.850
Hypertension	−0.21 (−1.11, 0.68)	0.640	−0.89 (−1.92, 0.15)	0.093	0.94 (−0.80, 2.68)	0.287
Diabetes	−1.28 (−2.34, −0.23)	0.018*	−0.85 (−2.17, 0.47)	0.204	−2.19 (−4.01, −0.38)	0.019*

FBG, fasting blood glucose; TG, triglyceride; TC, total cholesterol; HDL, high-density lipoprotein; LDL, low-density lipoprotein; VLDL, very low-density lipoprotein. Corrected P-value was 0.004 by Bonferroni correction. *P < 0.05.

To further identify the independent variables among the risk factors, multiple linear regression analysis was performed. After adjusting confounders, TG remained negatively associated with LTL in all patients [β(95%CI) = −0.69 (−1.26, −0.11), *P* = 0.019] (Table 4). Lack of exercise [β(95%CI) = −1.80 (−3.12, −0.49), *P* = 0.025] and TG levels [β(95%CI) = −3.22 (−6.05, −0.390), *P* = 0.026] were negatively associated with LTL in male patients, while dyssomnia [β (95%CI) = −2.00 (−3.96, −0.07), *P* = 0.045] and diabetes [β(95%CI) = −2.13 (−4.10, −0.27), *P* = 0.035] were negatively associated with LTL in female patients (Table 4). Cohen’s effect size *f*^2^ was reported for statistical power (Table 4).

**TABLE 4:** Multiple linear regression analysis for LTL.

Model	Variable	Multivariable linear regression analysis				
		All (*n* = 312)	Male (*n* = 208)	Female (*n* = 104)		
		β or β (95% CI)	β or β (95% CI)	β or β (95% CI)	*P*	*f* ^2^
1	TG	−0.69 (−1.26, −0.11)			0.019*	0.086
2	Lack of exercise		−1.80 (−3.12, −0.49)		0.025*	0.129
	LDL		−3.22 (−6.05, −0.390)		0.026*	
3	Sleeping status			−2.00 (−3.96, −0.07)	0.045*	0.176
	Diabetes			−2.13 (−4.10, −0.27)	0.035*	

*β, regression coefficient; CI, confidence interval; f^2^, Cohen’s effect size.*

*Predictors in Model 1 were for all stroke patients: adjusted for BMI, smoking status (former or current smokers/never smokers), alcohol drinking status (alcohol drinkers/never or occasionally alcohol drinkers), exercises status (well/poor), sleeping status (well/dyssomnia), HDL, LDL, VLDL, diabetes (yes/no), and hypertension (yes/no).*

*Predictors in Model 2 were for male stroke patients: adjusted for BMI, smoking status (former or current smokers/never smokers), alcohol drinking status (alcohol drinkers/never or occasionally alcohol drinkers), sleeping status (well/dyssomnia), TG, HDL, VLDL, diabetes (yes/no), and hypertension (yes/no).*

Predictors in Model 3 were for female stroke patients: adjusted for BMI, exercises status (well/poor), TG, HDL, LDL, and hypertension (yes/no). *P < 0.05.

## Discussion

Leukocyte telomere length is short in patients with stroke. Consistently, cardiovascular risk factors, also correlated with stroke, were associated with short LTL in a case-control study (Alzain et al., 2017). However, the heterogeneity of LTL is high in stroke patients. To minimize the age effect on the heterogeneity, we analyzed LTL in 73 healthy controls and 57 stroke patients within a narrow age range, 50–60 years. No significant difference was detected between healthy controls and patients. Then, the gender effect was investigated. Although the number of female patients is less, the variance of LTL in the female patients is very mild within 50–60 years (Lapham et al., 2015). Different trends were observed in male and female patients. LTL of controls was almost equal to that of male patients, while LTL tended to be short in female patients compared with controls (Supplementary Figure 1). It suggests that gender-specific effects on LTL may exist in stroke patients. Moreover, the importance of gender difference in the disease population has been investigated in a couple of studies (Sullivan et al., 2019). However, no study was performed on stroke patients. Thus, the gender difference of LTL should be investigated in patients with stroke.

In this study, significant differences in demographic characteristics, lifestyles, VLDL levels, and disease history were detected between male and female patients. From a lifestyle aspect, LTL was associated with exercise status in male patients and sleep quality in female patients. However, LTL was not associated with demographic characteristics, disease history, and medications in stroke patients. Importantly, lack of exercise and TG levels were negatively associated with LTL in male patients but not in female patients, while dyssomnia and diabetes were negatively associated with LTL in female patients but not in male patients by multiple regression analysis. Our finding clearly showed that gender difference does exist in associations between risk factors and LTL.

Triglyceride, an independent risk factor, was negatively associated with LTL in male patients but not in female patients. It was reported that high TG is associated with obesity and is a target for obesity treatment (Chen and Farese, 2000). Recent evidence suggests that excess adipose tissue plays a key role in inducing a chronic and systemic inflammatory state contributing to TL shortening in obese individuals (Welendorf et al., 2019). However, no studies have reported gender differences in obesity-associated TL shortening. In this study, we showed that LTL tended to be shorter in male patients than that in female patients when BMI > 28 although it was not significant. Moreover, weight loss has been shown to promote the improvement of chronic inflammation and oxidative stress of adipose tissue and alleviate telomere attrition. It suggests that obesity might be more harmful to men than to women, which might be associated with high TG levels in men. Lack of exercise was associated with short LTL in male patients but not in female patients. Lack of exercise is complemented by housework in female patients as females usually share more housework than males.

Previous reports suggest that women have at least a 40% increased risk of insomnia compared with men (Guidozzi, 2015). Gender differences in associations between sleep and LTL have not been investigated in any populations although associations between sleep duration, snoring or circadian rhythm disturbance, and LTL were reported (Barceló et al., 2010; Carroll et al., 2016; Shin et al., 2016; Wynchank et al., 2019). Our study showed that sleep quality was associated with LTL in female patients but not in male patients. Diabetes was independently associated with LTL in female patients but not in male patients. It may be associated with the dysregulation of endocrine and metabolism systems, which plays a more pivotal role in females than in males. Our study suggested that the quality of sleep and the well management of diabetes are more critical for female patients.

We acknowledge a number of limitations in this study. First, due to the nature of the cross-sectional study, no causal effect of risk factors on LTL shortening can be concluded although we provided gender differences in associations between risk factors and LTL. Second, our results may be affected by confounders not included in this study such as socioeconomic status and physical activity status in addition to exercises. Moreover, the sample size is limited, particularly for subgroup analysis. The statistical power ranges from a small effect in the total patient group to medium effects in male and female subgroups based on Cohen’s effect size *f*^2^. Although a number of trends were found in our study, they did not reach significance. In addition, racial difference is not considered as only Han Chinese patients were included in this study. The study needs to be improved by increasing sample size, including more factors associated with LTL, recruiting patients of different races, and conducting cohort studies in the future.

## Conclusion

In conclusion, our study showed that LTL is differently associated with risk factors in male and female patients with stroke. It indicated that gender difference should be considered when LTL is potentially applied as an index of risk and prognosis for stroke. Our study also provides an insight into that gender differences should be considered when developing intervention strategies for stroke prevention and treatment. However, a study with an increased sample size is still necessary.

## Data Availability Statement

The original contributions presented in the study are included in the article/Supplementary Material, further inquiries can be directed to the corresponding author/s.

## Ethics Statement

The studies involving human participants were reviewed and approved by Research Ethics Committee of Jining Medical University. The patients/participants provided their written informed consent to participate in this study. Written informed consent was obtained from the individual(s) for the publication of any potentially identifiable images or data included in this article.

## Author Contributions

YH and YW contributed to the study design. YQW and FJ performed the experiments, analyzed the data, and wrote the manuscript. RL, XZ, and LY contributed to the data collection and critical comments. HZ, QK, YH, and YW revised the manuscript. All authors reviewed and approved the manuscript.

## Conflict of Interest

The authors declare that the research was conducted in the absence of any commercial or financial relationships that could be construed as a potential conflict of interest.

## Publisher’s Note

All claims expressed in this article are solely those of the authors and do not necessarily represent those of their affiliated organizations, or those of the publisher, the editors and the reviewers. Any product that may be evaluated in this article, or claim that may be made by its manufacturer, is not guaranteed or endorsed by the publisher.
