# Supplementary material for: Gender Difference in Associations Between Telomere Length and Risk Factors in Patients With Stroke
Source: Front Aging Neurosci. 2021 Nov 4;13:719538. doi: 10.3389/fnagi.2021.719538 (PMC8600313; doi:10.3389/fnagi.2021.719538)
Supplement: Supplementary file 1 [file Data_Sheet_1.docx]

| **Table 1: Medication of stroke patients** | | | | |
| --- | --- | --- | --- | --- |
| Variables | Total (n=312) Number (%) | Male (n=208) Number (%) | Female (n=104) Number (%) | *P* (Male vs. Female) |
| Calcium channel blocker | 29 (9.30) | 19 (9.10) | 10 (9.60) | 0.520 |
| Biguanides | 23 (7.40) | 12 (5.80) | 11 (10.60) | 0.098 |
| Aspirin | 44 (14.1) | 26 (12.50) | 18 (17.3) | 0.164 |
| Clopidogrel | 12 (3.80) | 9 (4.30） | 3 (2.90) | 0.390 |
| Alpha glycosidase inhibitors | 8 (2.56) | 3 (1.44) | 5 (4.81) | - |
| Diuretic | 2 (0.64) | 2 (0.96) | 0 | - |
| Beta-receptor-blocking agent | 6 (1.92) | 3 (1.44) | 3 (2.88) | - |
| Glitazones | 3 (0.96) | 1 (0.48) | 2 (1.92) | - |
| Insulin | 6 (1.92) | 3 (1.44) | 3 (2.88) | - |
| Statins | 7 (2.24) | 6 (2.88) | 1 (0.96) | - |

| **Table 2: Difference of LTL based on medications** | | | | | | | | |
| --- | --- | --- | --- | --- | --- | --- | --- | --- |
| Variables |  | Total | | Male | | Female | | *P* |
|  |  | Median (IQR) | *P* | Median (IQR) | *P* | Median (IQR) | *P* | (Male vs. Female) |
| Calcium channel blocker | Yes | 2.41 (1.86,4.53) | 0.768 | 2.15 (1.69, 4.67) | 0.610 | 2.95 (2.31. 5.09) | 0.791 | 0.179 |
|  | No | 2.92 (1.71, 6.42) |  | 2.93 (1.64, 5.31) |  | 2.88 (1.78, 8.00） |  | 0.402 |
| Biguanides | Yes | 2.81 (1.68, 8.58) | 0.769 | 3.36 (1.73, 7.60) | 0.489 | 2.12 (1.26, 9.77) | 0.747 | 0.786 |
|  | No | 2.85 (1.76, 6.20) |  | 2.84 (1.64, 4.75) |  | 2.92 (1.86, 7.85) |  | 0.218 |
| Aspirin | Yes | 2.74 (1.86, 5.38) | 0.791 | 2.12 (1.45,7.48) | 0.510 | 3.10 (2.32, 3.92) | 0.810 | 0.294 |
|  | No | 2.89 (1.73, 6.39) |  | 12.90 (1.68, 4.69) |  | 2.88 (1.77, 8.70) |  | 0.418 |
| Clopidogrel | Yes | 2.39 (1.40, 6.36) | 0.508 | 3.35 (1.22, 8.23) | 0.775 | 2.38 | 0.400 | 0.864 |
|  | No | 2.86 (1.76, 6.29) |  | 2.83 (1/65, 4.72) |  | 3.05 (1.81, 7.95) |  | 0.246 |

| **Table 3. Pearson product-moment correlation analysis of LTL and multiple risk factors** | | | | | | |
| --- | --- | --- | --- | --- | --- | --- |
| Variable | All (n=312) | | Male (n=208) | | Female (n=104) | |
|  | *r* | *P* | *r* | *P* | *r* | *P* |
| Age | 0.047 | 0.413 | -0.013 | 0.847 | 0.102 | 0.304 |
| BMI | -0.055 | 0.335 | -0.079 | 0.260 | 0.140 | 0.155 |
| FBG | -0.115 | 0.049^*^ | -0.115 | 0.107 | -0.143 | 0.166 |
| TG | -0.134 | 0.020^*^ | -0.151 | 0.032^*^ | -0.142 | 0.159 |
| HDL | 0.020 | 0.728 | 0.094 | 0.185 | 0.058 | 0.573 |
| LDL | -0.110 | 0.058 | -0.148 | 0.036^*^ | -0.007 | 0.947 |
| VLDL | -0.014 | 0.819 | -0.043 | 0.550 | 0.020 | 0.850 |
| ^*^*P* < 0.05; *r:* correlation coefficient; FBG: fasting blood-glucose; TG: triglyceride; HDL: high-density lipoprotein; LDL: low density lipoprotein; VLDL: very low density lipoprotein. Corrected *P* value was less than 0.007 by Bonferroni-correction. | | | | | | |

**Figure 1**

**
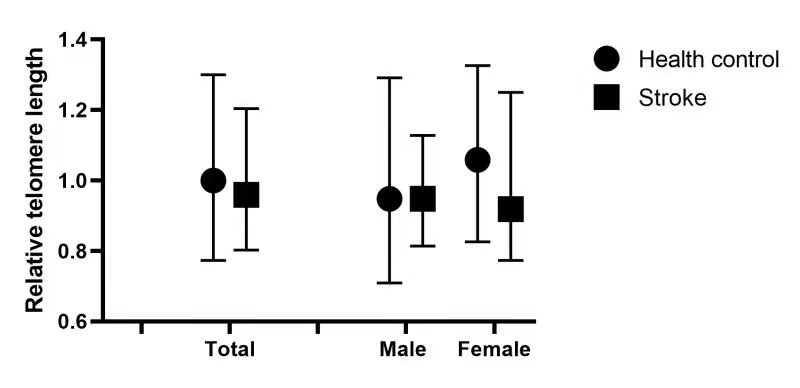
**

**Gender difference of relative LTL in healthy controls and patients with stroke aged 50-60y.** Values represent median with IQR. 73 controls (36 males and 37 females) and 57 patients (36 males and 21 females) were included. All numbers were relative to LTL of total health controls.
